# Miglitol, an Oral Antidiabetic Drug, Downregulates Melanogenesis in B16F10 Melanoma Cells through the PKA, MAPK, and GSK3β/β-Catenin Signaling Pathways

**DOI:** 10.3390/molecules28010115

**Published:** 2022-12-23

**Authors:** Hyeon-Mi Kim, Chang-Gu Hyun

**Affiliations:** Jeju Inside Agency and Cosmetic Science Center, Department of Chemistry and Cosmetics, Jeju National University, Jeju 63243, Republic of Korea

**Keywords:** B16F10, drug repurposing, melanogenesis, miglitol, signaling pathways

## Abstract

Hyperpigmentation is a common condition that causes darker spots or patches on the skin, which often look brown, black, gray, red, or pink. This results in unresolved psychological impact due to high anxiety, depression, and somatoform disorder. We aimed to repurpose an antidiabetic drug, miglitol, as an effective compound against hyperpigmentation when applied as a cosmeceutical agent. The present study investigated the antimelanogenic effects of miglitol and the trehalase inhibitor validamycin A. Miglitol in isolation exhibited no cytotoxicity and significantly reduced the melanin production and intracellular tyrosinase activity in B16F10 melanoma cells. The Western blotting results showed that miglitol reduces the expression of melanogenic regulatory factors, including tyrosinase, tyrosinase-related protein (TRP)-1, TRP-2, and microphthalmia-associated transcription factor (MITF). Mechanistically, miglitol appears to suppress melanin synthesis through cAMP-dependent protein kinase (PKA)-dependent downregulation of MITF, a master transcription factor in melanogenesis. The antimelanogenic effects of miglitol was mediated by downregulation of the p38 signaling pathway and upregulation of extracellular signal-regulated kinase (ERK). Moreover, miglitol decreases P-GSK3β and β-catenin levels compared to those in the untreated group. However, miglitol activated P-β-catenin expression compared to that in the untreated group. Finally, we tested the potential of miglitol in topical application through primary human skin irritation tests on the normal skin (upper back) of 33 volunteers. In these assays, miglitol (125 and 250 μM) did not induce any adverse reactions. Taken together, these findings suggest that the regulation of melanogenesis by miglitol may be mediated by the PKA, MAPK, and GSK3β/β-Catenin signaling pathways and that miglitol might provide new insights into drug repurposing for the treatment of hyperpigmentation symptoms.

## 1. Introduction

Drug repurposing is broadly defined as a drug development strategy, wherein the use of an already approved drug is proposed for a new indication. In this process of establishing a new drug, the category of existing approved drugs is targeted for discovering new effects and/or targets. This approach has several advantages for drug development [1]. First, the toxicity of repurposed drugs is likely to be minimal compared with newly developed drugs, because they have been demonstrated to be sufficiently safe in preclinical or phase 1 studies. Therefore, most tolerability studies, such as safety assessment, dose optimization, and route determination, can be bypassed or minimized. Second, it is possible to reduce the redevelopment investment by using the established large-scale production process and quality standards or manufacturing process test methods.

Although many small-molecule inhibitors and activators of human diseases have been identified via drug repurposing, for some skin diseases like hyperpigmentation, developing effective antimelanogenic agents via drug repurposing remains challenging. Herein, we examined the applicability of drug repositioning of the FDA-approved antidiabetic drug miglitol for treating hyperpigmentation.

**Figure 1:**
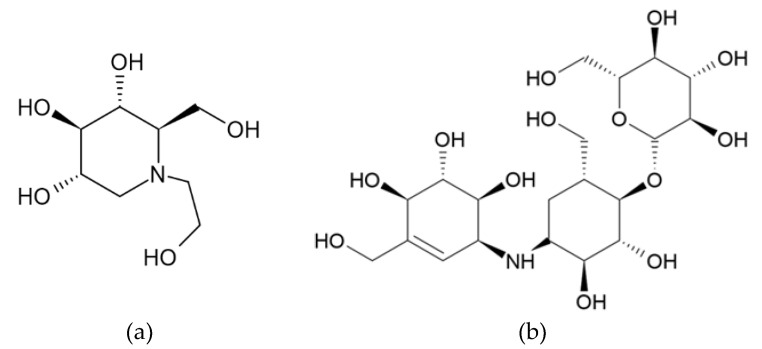
Chemical structure of (**a**) miglitol and (**b**) validamycin A.

As shown in Figure 1, an α-glucosidase inhibitor is a structural warhead of various natural and synthetic compounds exhibiting various pharmacological activities. Its derivatives are attracting attention because of their oral antidiabetic, anticoronavirus (COVID-19), and antibiotic properties in combination with fungistatic effects [2,3,4]. There has been constant interest in the synthesis of these compounds. Miglitol is a widely used second-generation semisynthetic α-glucosidase inhibitor derived from 1-deoxynojirimycin that is structurally similar to glucose and effective in the treatment of type 2 diabetes mellitus (T2DM) [5]. Miglitol is generally prescribed to diabetics because it inhibits α-glucosidase in small intestines, reducing postprandial hyperglycemia and prolonging carbohydrate absorption. Miglitol was approved as an antidiabetic drug in 1996, and there is growing evidence that miglitol also exerts an anti-obesity effect based on both animal and human studies [6]. In addition, miglitol attenuates glucose fluctuation, heart rate variability, and sympathetic activity in patients with type 2 diabetes and acute coronary syndrome [7]. Furthermore, miglitol increases hepatic cholesterol 7 alpha-hydroxylase (CYP7A1) activity in association with altered short-chain fatty acid production in the gut of obese diabetic mice [8]. Interestingly, miglitol also attenuates non-alcoholic steatohepatitis in diabetic patients [9].

As mentioned above, drug repositioning provides a quick way to identify biologically active ingredients with proven safety profiles required for developing cosmeceuticals. Therefore, we focused on the FDA-approved antidiabetic miglitol in our efforts to find a new application with potent yet safe skin health effects. In the present study, we designed a novel approach to elucidate the antimelanogenic properties of miglitol through regulation of the PKA/CREB, MAPK, and Wnt/β-catenin signaling pathways in a B16F10 cell model. Furthermore, we tested the potential application of miglitol as a topical antimelanogenic agent via human skin primary irritation tests.

## 2. Results

### 2.1. Miglitol Alone Inhibits Melanin Production and Tyrosinase Activity in B16F10 Melanoma Cells

**Figure 2:**
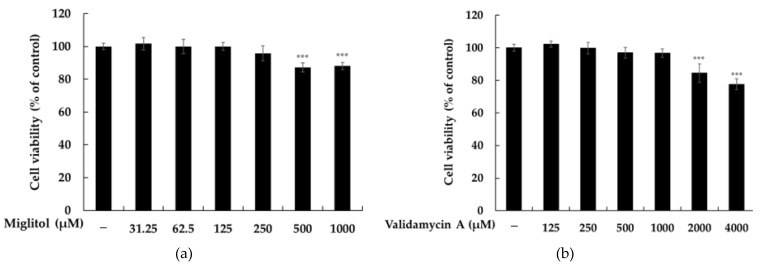
Viability of α-glucosidase inhibitors in B16F10 melanoma cells. Cell viability of (**a**) miglitol (31.25–1000 µM) and (**b**) validamycin A (125–4000 µM) was investigated by MTT assay. The cells were plated at 1.5 × 10^4^ cells/well in 24-well plates for 24 h, and each sample was treated at various concentrations for 72 h. Cell viability is expressed as percentages relative to non-treated cells. Data are presented as mean ± standard deviation (SD) of at least three independent experiments (*n* = 3). *** *p* < 0.001 vs. the non-treatment control.

**Figure 3:**
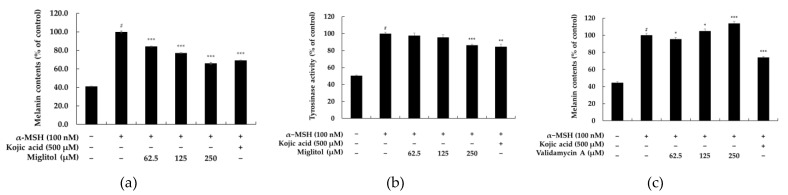
Effects of α-glucosidase inhibitors on the melanin content and tyrosinase activity in α-MSH-stimulated B16F10 cells. Cells were plated in a 60 mm cell culture dish (8.0 × 10^4^ cells/dish) and incubated for 24 h, followed by (**a**,**b**) miglitol (62.5–250 µM) and (**c**) validamycin A (62.5–250 µM) pre-treatment for 1 h and subsequent treatment with α-melanocyte-stimulating hormone (α-MSH, 100 nM) for 72 h. The cells were then harvested and assayed. α-MSH was used as the negative control, and kojic acid (500 µM) was used as the positive control. Melanin content and tyrosinase activity data are presented as the mean ± standard deviation (SD) of at least three independent experiments (*n* = 3). # *p* < 0.001 compared to the non-treatment group; * *p* < 0.05, ** *p* < 0.01, and *** *p* < 0.001 vs. the α-MSH only group.

The chemical structures of miglitol and validamycin A are shown in Figure 1. We evaluated the cytotoxicity of miglitol and validamycin A against B16F10 cells and found that application of either miglitol or validamycin A did not exert any toxicity up to concentrations of 250 and 1000 μM (Figure 2). Therefore, we conducted further experiments at concentrations lower than 250 μM. As shown in Figure 3a, miglitol demonstrated a concentration-dependent reduction in melanin content comparable to that of 500 μM of kojic acid, a positive control known to exhibit strong whitening effects. Consistently, there was decreased tyrosinase activity in cells pretreated with miglitol (Figure 2b), whereas validamycin A treatment had no effect on melanin production in B16F10 cells. Therefore, further experiments were performed to evaluate the melanogenesis effects of miglitol.

### 2.2. Miglitol Inhibits the Expression of Melanogenesis-Related Proteins

**Figure 4:**
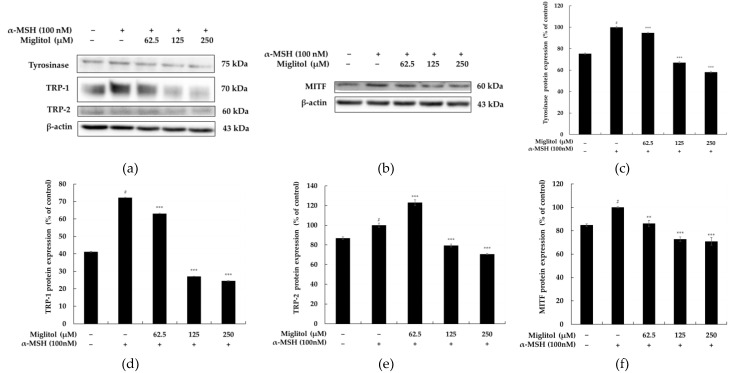
The effect of miglitol on tyrosinase, TRP-1, TRP-2, and MITF protein levels in α-MSH-stimulated B16F10 cells. Cells were treated with miglitol (62.5, 125, and 250 μM) for 48 h in the presence of α-MSH (100 nM). (**a**,**b**) Western blotting results and (**c**) tyrosinase, (**d**) TRP-1, (**e**) TRP-2 protein expression, and (**f**) MITF protein expression. α-MSH was used as the negative control. The results are presented as the mean ± SD from three repeated measurements using ImageJ. # *p* < 0.001 vs. the unstimulated control group; ** *p* < 0.01 and *** *p* < 0.001 vs. α-MSH alone.

Melanin synthesis requires three important melanogenic enzymes: tyrosinase, tyrosine-related protein (TRP)-1, and TRP-2. Most importantly, the microphthalmia-associated transcription factor (MITF) acts as a master regulator for the expression of these three enzymes. To explore the mechanism underlying the inhibition of melanogenesis activity by miglitol, we characterized the effects of miglitol on tyrosinase, TRP-1, TRP-2, and MITF in B16F10 cells by Western blotting. As shown in Figure 4, the protein levels of tyrosinase, TRP-1, and TRP-2 were significantly reduced by miglitol compared with the untreated control group. In addition, MITF expression was also reduced by miglitol. These results clearly suggest that miglitol inhibits melanogenesis through the MITF-mediated downregulation of tyrosinase, TRP-1, and TRP-2.

### 2.3. Miglitol Inhibits Melanogenesis through the PKA Signaling Pathway

**Figure 5:**
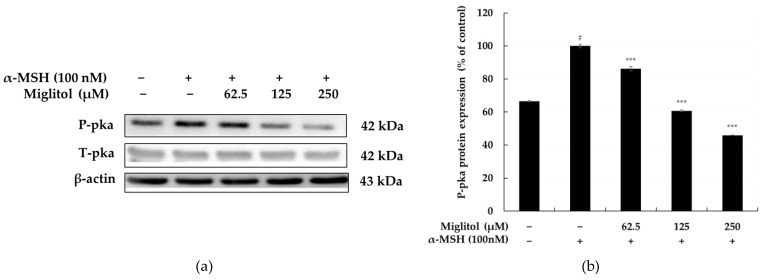
The effect of miglitol on PKA protein expression in α-MSH-stimulated B16F10 cells. Cells were treated with miglitol (62.5, 125, and 250 μM) for 24 h in the presence of α-MSH (100 nM). (**a**) Western blotting results, (**b**) P-PKA protein expression. α-MSH was used as the negative control. The results are presented as the mean ± SD from three repeated measurements using ImageJ. # *p* < 0.001 vs. the unstimulated control group; *** *p* < 0.001 vs. α-MSH alone.

The PKA signaling-mediated expression of the MITF gene sequentially upregulates the expression of tyrosinase, TRP-1, and TRP-2, critical factors in melanogenesis. Therefore, after demonstrating that miglitol could induce the expression of MITF, tyrosinase, TRP-1, and TRP-2, we used Western blotting to further determine whether PKA is involved in the melanogenic activity of miglitol through PKA signaling in B16F10 cells. As shown in Figure 5, miglitol treatment significantly downregulated the expression level of phosphorylated PKA compared with the control treatment. Thus, the above findings indicate that miglitol suppresses MITF expression through downregulation of the PKA/CREB signaling pathways, leading to a decrease in melanogenesis.

### 2.4. Miglitol Modulates Melanogenesis via the MAPK Signaling Pathway

**Figure 6:**
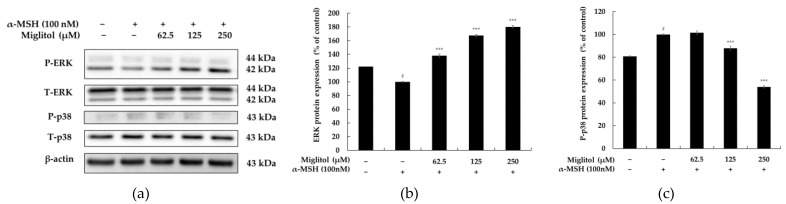
The effect of miglitol on MAPK protein expression in α-MSH-stimulated B16F10 cells. Cells were treated with miglitol (62.5, 125, and 250 μM) for 4 h in the presence of α-MSH (100 nM). (**a**) Western blotting results and (**b**) p-ERK, and (**c**) p-p38 protein expression. α-MSH was used as the negative control. The results are presented as the mean ± SD from three repeated measurements using ImageJ. # *p* < 0.001 vs. the unstimulated control group; *** *p* < 0.001 vs. α-MSH alone.

It has been observed in previous research that MITF expression is controlled by phosphorylation of mitogen-activated protein kinases (MAPKs). Moreover, it has also been shown that inhibition of p38 and c-Jun N-terminal kinase (JNK), along with activation of extracellular signal-regulated kinase (ERK) phosphorylation, reduces MITF and melanogenic enzyme expression, resulting in the downregulation of melanogenesis [10,11]. Therefore, we evaluated the phosphorylation of p38 and ERK MAPKs to investigate the upstream cascade related to the antimelanogenesis effect of miglitol. As shown in Figure 6, miglitol treatment remarkably increased the level of phosphorylated ERK and significantly reduced p38 phosphorylation compared with the control treatment. These results suggest that miglitol exerts antimelanogenic effects in B16F10 cells by reducing MITF expression via the MAPK signaling pathway.

### 2.5. Miglitol Modulates Melanogenesis via the GSK3β/β-Catenin Signaling Pathway

**Figure 7:**
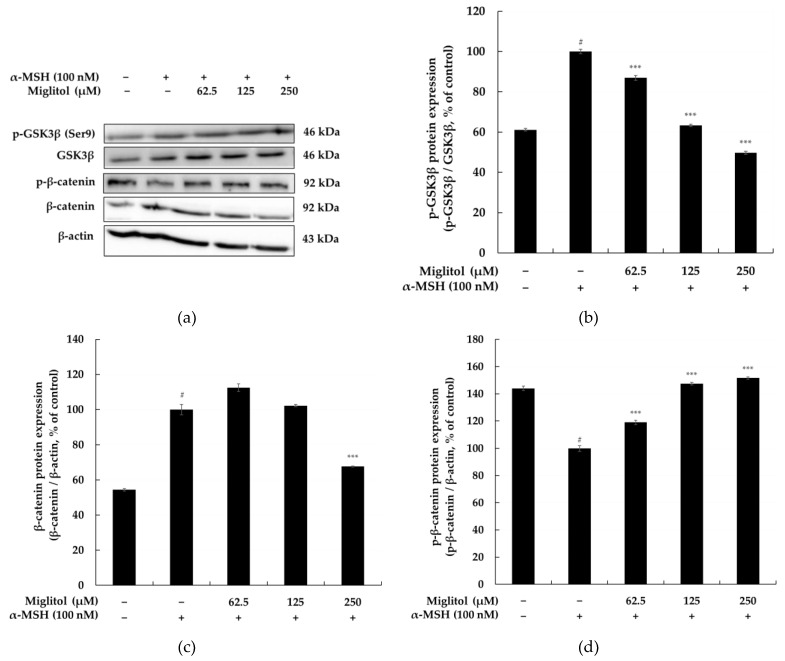
Effects of miglitol on the Wnt/β-catenin signaling pathway in α-MSH-induced B16F10 melanoma cells. (**a**) Western blotting results and (**b**) GSK3β, (**c**) β-catenin, and (**d**) p-β-catenin protein expression. Western blot analyses of the dose-dependent effect of miglitol (62.5, 125, and 250 μM) on the expression levels of GSK3β, β-catenin, and p-β-catenin in B16F10 cells treated with α-MSH (100 nM) for 24 h. Equal amounts of protein loading were confirmed using β-actin. Data are expressed as the mean SD from a single triplicate experiment using ImageJ software. # *p* < 0.001 vs. the untreated control group; *** *p* < 0.001 vs. α-MSH alone.

GSK3 (Ser 9) phosphorylated through the Wnt/β-catenin pathway has been reported to induce β-catenin accumulation in the cytoplasm; the accumulated β-catenin is transferred to the nucleus to increase MITF expression [10]. We investigated whether miglitol inhibits melanogenesis through the Wnt/β-catenin signal in B16F10 cells. The results showed that miglitol decreases P-GSK3β (Ser 9) and β-catenin compared to those in the untreated group. However, miglitol activated P-β-catenin expression compared to that in the untreated group. These results suggest that miglitol decreases melanogenesis through the Wnt/β-catenin signaling pathway (Figure 7).

### 2.6. Safety of Miglitol Demonstrated through Human Primary Irritation Tests

The potential of miglitol in topical applications was then tested by primary human skin initiation tests. Miglitol was applied to the skin area at concentrations of 125 and 250 µM for up to 24 h. Thereafter, after removing miglitol, the patch corresponding to the application area was observed for 20 min and 24 h. Squalene (solvent) was used as a negative control. Miglitol was classified as causing “no to slight irritation” in terms of the primary irritation potential on human skin, as shown in Table 1.

## 3. Discussion

Tyrosinase is a type 1 membrane-bound glycoprotein that catalyzes the initial and rate-limiting steps of melanin production in its ultimate home, the melanosome [12]. Tyrosinase activity requires N-glycan processing of tyrosinase, which is performed by intracellular α-glucosidase and α-mannosidases in the endoplasmic reticulum and Golgi apparatus for functionality and proper localization. If these enzymes are inhibited, tyrosinase is aberrantly folded and does not become mature, resulting in hypopigmentation [13]. Therefore, many studies have reported on whether α-glucosidase inhibitors can be used as melanogenic inhibitors [14,15,16,17]. In this study, we aimed to elucidate whether the antidiabetic drug miglitol is an effective inhibitor in melanin production and can thus be repurposed as a cosmeceutical agent.

In the past decade, drug repurposing has generated explosive interest as an explicit drug development strategy and because it offers advantages over traditional methods. Drug repurposing is broadly defined as a drug development strategy in which the use of an already approved drug for a new indication is proposed. In this process, new effects and/or targets for the approved new drug are discovered and further targeted in the category of previously approved drugs [1]. In this study, we tried to find new effects and/or targets for miglitol, an antidiabetic agent, via a drug repurposing strategy and first confirmed the applicability to hyperpigmentation.

Drug discovery in skin drug therapy is a huge and continuously expanding field. Scientists are developing new and sensitive medicines and drugs that target specific receptors to produce a consistent and appropriate response. Melanin is a major target of skin drug therapy and is synthesized in a special cell organ called the melanosome, which plays an essential role in protecting the skin from harmful sunlight under normal conditions. However, increased melanin production and excessive accumulation can lead to various skin problems, such as freckles and spots [18]. Abnormal pigmentation is a major concern causing serious aesthetic problems, especially among female populations, and skin-lightening agents—including arbutin, kojic acid, and ellagic acid—are widely used for treatment, especially in Asian countries. However, many of the currently widely used depigmenting agents are shown to be toxic to melanocytes and to cause adverse effects. Kojic acid has been banned as a cosmetic ingredient in many countries because it has been demonstrated to cause hepatocellular toxicity, skin cancer, and contact dermatitis in numerous clinical trials. Therefore, attention has been focused on alternative strategies, such as the repurposing of drugs that have already been proven to be safe and effective as a supplement to treat some disorders in humans [19].

We aimed to repurpose an antidiabetic drug as an effective compound against hyperpigmentation and apply it as an ingredient in cosmeceutical agents. The present study focused on investigating the antimelanogenic effects of the antidiabetic drug miglitol (Figure 1). Mouse melanoma B16F10 cells are highly pigmented cells that share the most similar melanogenic mechanisms with human epidermal melanocytes and, thus, are commonly used in in vitro assays of melanogenic inhibitors. Therefore, in this study, we investigated the effects of miglitol and validamycin A on melanogenesis and their molecular mechanisms to understand the underlying signaling pathways in B16F10 cells. Clinical trials with miglitol (typically 50 or 100 mg, three times a day) in patients with type 2 diabetes consistently demonstrated significant improvement in blood glucose control over 6 to 12 months. Considering that the molecular weight of miglitol is 207.2 g/mol, its dose corresponds to 250 μM to 500 μM. The results of the current study demonstrate that, within the safe concentration range (62.5–250 μM), significant suppression of melanin synthesis by miglitol, and not validamycin A, occurs by inhibiting cellular tyrosinase activity without having any cytotoxic effects on B16F10 cells (Figure 3). Therefore, further experiments were performed to explore the potential molecular mechanisms of miglitol that act on the protein expression levels of important pathways regarding melanogenesis in B16F10 cells.

Melanogenesis is a complex process that involves enzymatic and chemical reactions. There is a “Yin and Yang” role for melanin and active melanogenesis in melanoma development, progression, and therapy. Interestingly, while eumelanin is believed to provide radioprotection and photoprotection by acting as an efficient antioxidant and sunscreen, pheomelanin, being less photostable, can generate a mutagenic environment after exposure to the short-wavelength UVR. Melanogenesis by itself and its highly reactive intermediates show cytotoxic, genotoxic, and mutagenic activities, and it can lead to melanoma progression and resistance to immunotherapy [20,21]. Therefore, Slominski et al. [20] emphasized that inhibition of melanogenesis in advanced melanotic melanoma is a realistic adjuvant strategy to enhance immuno-, radio-, and chemotherapy. The tyrosinase family, which consists of tyrosinase, TRP-1, and TRP-2, is the most critical regulator of melanogenesis. Our data indicate that the miglitol concentration dependently decreased melanin content and intracellular tyrosinase activity in α-MSH-induced B16F10 cells. In addition, we found that miglitol-reduced melanogenesis was associated with the decreased protein expression of tyrosinase, TRP-1, and TRP-2 (Figure 4). MITF increases the expression of tyrosinase, TRP-1, and TRP-2 by binding to the M-box shared by the three genes in their promoter regions, and, accordingly, melanin synthesis in melanocytes increases as a result of MITF activation. Both genetic mutations and exogenous stimuli can alter MITF expression levels, consequently leading to dysregulation and pigmentation disorders in the melanogenesis processes. Therefore, MITF often serves as the target transcription factor in screening for inhibitors of melanin production. Our data show that miglitol treatment inhibits MITF expression in mouse B16F10 cells, thereby leading to a decrease in intracellular tyrosinase activity and melanin production. These observations suggest that miglitol augments the functionality of MITF signaling-dependent melanin biosynthesis in B16F10 cells. It is notable that the higher inhibitory potency on melanin production and tyrosinase activity were demonstrated by miglitol than by the well-known skin-whitening agent kojic acid (Figure 3). Furthermore, we also unveiled the possible molecular mechanisms by which miglitol inhibits melanogenesis using inhibitors of the PKA, MAPK/ERK, and p38 MAPK signaling pathways.

Studies have shown that α-MSH binds directly to its receptor MC1R and activates adenylate cyclase to increase cAMP levels, activate PKA, and continuously activate CREB phosphorylation/activation in melanocytes, ultimately promoting binding to MITF promoters [10,11]. As expected, our data clearly show that miglitol treatment inhibits phosphorylation of PKA in α-MSH-stimulated B16F10 cells, indicating that miglitol-mediated MITF downregulation is mediated by the inhibition of the α-MSH-induced PKA pathway (Figure 5).

MAPK family members, including ERK, JNK, and p38 MAPK, are key signaling molecules involved in the regulation of melanogenesis. Studies have shown that ERK phosphorylation leads to a downregulation in melanin synthesis. In contrast, the phosphorylation of JNK and p38 MAPK activates MITF to ultimately stimulate melanogenesis. Nevertheless, the overall role of MAPK pathway activation in melanin production remains controversial. In B16F10 cells, most melanogenic inhibitors suppress melanin production by decreasing the levels of p38 MAPK phosphorylation [10,11]. Meanwhile, it has been demonstrated that activation of p38 MAPK levels through fenofibrate, resorcinol, and fermented unpolished black rice suppresses melanogenesis [22,23,24]. In addition, studies have shown that increasing p-ERK levels inhibits melanin biosynthesis. In contrast, schisandrin B suppresses melanogenesis by decreasing the levels of ERK phosphorylation [25]. Therefore, we determined the effect of miglitol on the activation of MAPK signaling pathways to further explore the molecular mechanisms of melanin synthesis in B16F10 cells. Our results indicate that miglitol inhibits melanogenesis by activating the ERK signaling pathway and suppressing the p38 MAPK signaling pathway, followed by downregulation of melanogenic proteins (Figure 6).

Previous studies have shown that both MITF and β-catenin are mediators of Wnt signaling during melanocyte differentiation and that β-catenin directly interacts with MITF to activate MITF-specific target genes. Inhibition of GSK3-mediated β-catenin phosphorylation has been reported as a key event in the Wnt-β-catenin signaling pathway [10]. Therefore, we used Western blotting to confirm involvement of the GSK3β/β-catenin signaling pathway in the suppression of melanogenesis by miglitol. As shown in Figure 7, the level of phospho-GSK3β was downregulated in response to miglitol, leading to GSK3β activation, which resulted in decreased β-catenin expression and increased phosphorylation.

Finally, we evaluated whether miglitol could potentially be applied as a topical ingredient using primary human skin irritation tests. To determine whether stimulation or sensation potential was present, miglitol at concentrations of 125 or 250 µM was tested in the normal skin (upper back) of 33 volunteers. In this analysis, miglitol was judged to cause “no to slight irritation”, confirming that it is a safe substance (Table 1). These results suggest that miglitol could prevent the pathogenesis of pigmentation disorders when used as a topical agent. 

In summary, these data show that miglitol suppresses melanogenesis by inhibiting the expression of MITF, which is a transcriptional regulator that is involved in the expression of other melanogenic proteins, including tyrosinase, TRP-1, and TRP-2. In addition, we found that the miglitol-reduced expression of MITF is dependent on the activation of the signaling pathways that involve downregulation of p-p38, p-PKA, GSK3β, and β-catenin and upregulation of p-ERK and p-β-catenin. Accordingly, we propose that miglitol may be used as a topical therapeutic agent to treat hyperpigmentation and a useful ingredient in skincare products to prevent skin darkening. However, the possible involvement of other mechanisms, such as the CREB and PI3K/AKT signaling pathways in inhibiting melanin synthesis through miglitol, still needs to be investigated in the future. In addition, the relative effectiveness of miglitol in normal human melanocytes remains to be determined in future studies. Furthermore, the efficacy and safety of miglitol-treated melanogenesis inhibition must be evaluated in animal and human models. 

## 4. Materials and Methods

### 4.1. Materials

The miglitol used in this study was purchased from TCI (Tokyo, Japan). For the cell culture, Dulbecco’s modified Eagle’s medium (DMEM) and penicillin–streptomycin (P/S) were purchased from Thermo Fisher Scientific (Waltham, MA, USA) and fetal bovine serum (FBS) from Merck Millipore (Burlington, MA, USA). α-Melanocyte-stimulating hormone (α-MSH), protease/phosphatase inhibitor cocktail, sodium hydroxide (NaOH), L-DOPA, lipopolysaccharide (LPS), and Griess reagent used for the cell experiments were purchased from Sigma-Aldrich (St. Louis, MO, USA). 3-(4,5-Dimethylthiazol-2-yl)-2,5-diphenyltetrazolium bromide (MTT), dimethyl sulfoxide (DMSO), phosphate-buffered saline (PBS), Tris-buffered saline (TBS), sodium dodecyl sulfate (SDS), radioimmunoprecipitation assay (RIPA) buffer, and enhanced chemiluminescence (ECL) kits were purchased from Biosesang (Seongnam, Gyeonggi-do, Republic of Korea). A BCA protein assay kit and 0.5% trypsin-ethylenediaminetetraacetic acid (10×) were purchased from Thermo Fisher Scientific (Waltham, MA, USA), and Tween 20 and 2× Laemmli sample buffer were obtained from Bio-Rad (Hercules, CA, USA). Skimmed milk was purchased from BD Difco (Sparks, MD, USA). Primary antibodies tyrosinase, TRP-1, TRP-2, MITF used for Western blotting were purchased from Santa Cruz Biotechnology (Dallas, TX, USA). p-ERK, ERK, p-p38, p38, p-JNK, JNK, p-PKA (Cat. 4781), PKA (Cat. 4782), p-GSK-3β, GSK-3β, p-β-catenin, β-catenin, β-actin, and secondary antibodies anti-mouse and anti-rabbit were purchased from Cell Signaling Technology (Danvers, MA, USA).

### 4.2. Cell Culture

B16F10 mouse melanoma cells were purchased from ATCC: The Global Bioresource Center (Manassas, VA, USA). The cells were cultured in Dulbecco’s modified Eagle’s medium (DMEM) with 10% fetal bovine serum (FBS) and 1% penicillin–streptomycin at 37 °C in a humidified 5% CO_2_ atmosphere.

### 4.3. MTT Assay

Cytotoxicity was assessed using an MTT assay. Cultured B16F10 cells (1.5 × 10^4^ cells/well) were treated with α-glucosidase inhibitors (a) miglitol (31.25–1000 μM) and (b) validamycin A (125–4000 μM) in 24-well plates and incubated for 72 h. For the MTT assay, the culture medium was replaced with 0.5 mg/mL of MTT (1 mL). The cells were incubated at 37 °C for 4 h; then, the medium was removed, and the formazan product was dissolved in dimethylsulfoxide. Absorbance was measured at 540 nm using a microplate reader (BioTek; Winooski, VT, USA).

### 4.4. Measurement of Melanin Content

B16F10 cells (8.0 × 10^4^ cells/dish) were incubated in 60 mm cell culture dishes for 24 h. α-Glucosidase inhibitors (a) miglitol (62.5, 125, and 250 μM) and (b) validamycin A (62.5, 125, and 250 μM) were used to pre-treat cells for 1 h first, followed by α-MSH (100 nM) treatment with culture for 72 h. Kojic acid (500 μM) was used as a positive control. The cells were washed with 1× cold PBS, and lysis buffer (RIPA buffer, 1% protease inhibitor cocktail) was added for lysis at 4 °C for 20 min. After centrifugation for 20 min at 15,000 rpm and −8 °C, the supernatant was removed to obtain a pellet. Cell pellets were dissolved in 1 N NaOH supplemented with 10% DMSO at 80 °C for 10 min. Absorbance was measured at 405 nm using a microplate reader (BioTek; Winooski, VT, USA).

### 4.5. Measurement of Tyrosinase Activity

Tyrosinase activity was estimated by measuring the rate of L-DOPA oxidation. B16F10 cells (8.0 × 10^4^ cells/dish) were incubated in 60 mm cell culture dishes for 24 h. α-Glucosidase inhibitors (a) miglitol (62.5, 125, and 250 μM) and (b) validamycin A (62.5, 125, and 250 μM) were used to pre-treat cells for 1 h first, followed by α-MSH (100 nM) treatment with culture for 72 h. Kojic acid (500 μM) was used as a positive control. The cells were washed with 1× cold PBS, and lysis buffer (RIPA buffer, 1% protease inhibitor cocktail) was added for lysis at 4 °C for 20 min. Afterward, centrifugation for 20 min at 15,000 rpm and −8 °C was conducted to obtain supernatants. The protein concentration was quantified at 20 μg/mL using a BCA protein assay kit. L-DOPA (2 mg/mL) was added to the quantified protein and incubated at 37 °C for 2 h. Absorbance was measured at 490 nm using a microplate reader (BioTek; Winooski, VT, USA).

### 4.6. Western Blot Analysis

For the loading sample, protein (30 μg/mL) and 2× Laemmli sample buffer were mixed at a 1:1 ratio and heated at 100 °C for 5 min. Samples were electrophoresed with SDS-polyacrylamide gel to separate the proteins by size. After transfer to PVDF membrane, the protein was blocked in 5% skimmed milk dissolved in TBS-T (Tris-buffered saline with 1% Tween 20) for 2 h. The membrane was washed with 1× TBS-T and the primary antibody, dissolved at a ratio of 1:2000, and allowed to react overnight with incubation at 4 °C. After washing the antibody, the secondary antibody, dissolved at a ratio of 1:1000, was reacted at room temperature for 2 h. After washing the antibody, the protein was expressed using an ECL kit and developed using Chemidoc (WL, Vilber Lourmat, Paris, France).

### 4.7. Primary Skin Irritation Test

Overall, this study included 33 volunteers (healthy women) between the ages of 21 and 54 years (mean age = 43.58 ± 9.29 years) who had never experienced irritant and/or allergic contact dermatitis. As a negative control, the solvent squalene was used. Miglitol dissolved in squalene was prepared and applied at concentrations of 125 and 250 μM. Primary skin irritation responses were assessed according to the PCPC guidelines. The reaction results for squalene and miglitol in squalene were calculated from the formula shown below. This study was approved by the Industrial Review Board (IRB) of Dermapro Inc. and was conducted in accordance with the Declaration of Helsinki, regarding the ethical principles for medical research, after obtaining written consent from each volunteer (IRB number: 1-220777-A-N-01-DICN22201).

(1) $$\mathrm{Response}=\frac{\sum \left(Grade\times No.\;of\;Responders\right)}{4\;\left(Maximum\;Grade\right)\times n\;\left(Total\;Subjects\right)}\times 100\times 1/2$$

### 4.8. Statistical Analyses

The results of the experiments are expressed as the mean and standard deviation (mean ± SD) through three repeated experiments. Statistical significance is expressed as a *p*-value using Student’s *t*-test: # *p* < 0.001 vs. the unstimulated control group; * *p* < 0.05, ** *p* < 0.01, and *** *p* < 0.001 vs. α-MSH or LPS alone.

## Figures and Tables

**Table 1:** The results from the primary human skin irritation tests (*n* = 33).

No.	Test Sample	No. of Respondents	20 min after Removal				24 h after Removal				Reaction Grade (R) *		
			+1	+2	+3	+4	+1	+2	+3	+4	24 h	48 h	Mean
1	Miglitol (125 μM)	0	-	-	-	-	0	-	-	-	0	0	0
2	Miglitol (250 μM)	0	-	-	-	-	-	-	-	-	0	0	0.0
3	Squalene	0	-	-	-	-	-	-	-	-	0	0	0

The reactions were assessed at 20 min and 24 h after the removal of the treatment by the investigator, according to the PCPC guidelines (2014). * The range of irritation from “no” to “slight irritation”: 0.00 ≤ *R* < 0.87.

## Data Availability

Not applicable.

## Sample Availability

The samples are not available from the authors.
